# Changes in objectively measured physical activity after a multidisciplinary lifestyle intervention in children with abdominal obesity: a randomized control trial

**DOI:** 10.1186/s12887-019-1468-9

**Published:** 2019-04-04

**Authors:** Lydia Morell-Azanza, Ana Ojeda-Rodríguez, Amaia Ochotorena-Elicegui, Nerea Martín-Calvo, María Chueca, Amelia Marti, Cristina Azcona-San Julian

**Affiliations:** 1 Department of Nutrition, Food Science and Physiology, University of Navarra, Irunlarrea 1, 31008, Pamplona, Navarra Spain; 2 Paediatric Endocrinology Unit, Department of Pediatrics, Clínica Universidad de Navarra, Pamplona, Spain; 3 IdiSNA (Navarra Institute for Health Research), Pamplona, Spain; 4 Department of Preventive Medicine & Public Health, School of Medicine, University of Navarra, Pamplona, Spain; 5 Center of Biomedical Research in Physiopathology of Obesity and Nutrition (CIBEROBN), Institute of Health Carlos III, Madrid, Spain; 6 Paediatric Endocrinology Unit, Complejo Hospitalario de Navarra, Pamplona, Spain

**Keywords:** Obesity children, MVPA, Accelerometer, Metabolic risk, Leptin

## Abstract

**Background:**

Physical activity (PA) is associated with changes in body composition that affect insulin sensitivity and leptin levels. Few studies have assessed the effect of lifestyle interventions on changes in objectively measured PA levels in obese children. To evaluate the effects of a multidisciplinary lifestyle intervention on anthropometric indices, biochemical parameters and accelerometer measured PA in abdominal obese children.

**Methods:**

A randomized control trial was performed in 106 children and adolescents with abdominal obesity. Participants were randomly assigned to usual or intensive care group for 8-week. PA was measured by accelerometry over four days including, at least, two weekdays in all participants. Both groups were encouraged to accumulate an extra time of 200 min per week in their PA.

**Results:**

At baseline, 75% of subjects do not fulfill the WHO recommendation of being more than 60 min/day on moderate-to-vigorous PA (MVPA). The intensive care group achieved a significant reduction in anthropometric indexes compared to the usual care but no significant change was found in biochemical or PA parameters. Both groups achieved a significant reduction in light PA. Interestingly, intensive care participants significantly increased MVPA in 5.5 min/day. Moreover, an inverse association between changes in MVPA and leptin levels was found.

**Conclusion:**

The two lifestyle intervention reduced anthropometric indexes and lowered light PA in abdominal obese children. No significant differences were observed between intensive care and usual care in regard to PA. Intensive care participants significantly increase physical activity (MVPA) and, changes in MVPA were inversely associated with changes in leptin levels after the intervention.

**Trial registration:**

ClinicalTrials.gov, Identifier: NCT03147261. Registered 10 May 2017. Retrospectively registered.

**Electronic supplementary material:**

The online version of this article (10.1186/s12887-019-1468-9) contains supplementary material, which is available to authorized users.

## Background

Dyslipidemia, hypertension, insulin resistance or type 2 diabetes, are the main alterations that derive from obesity and contribute to aggravate cardiometabolic risk in pediatric populations [1]. Obese children with waist circumference at/or above the 90th percentile are at higher risk for dyslipidemia and insulin resistance than obese children with normal waist circumference [2, 3]. The factors that contribute to childhood obesity are complex, and include an excessive energy intake, a decrease in physical activity and an increase in sedentary behaviors, for example, the screen-time activities [4].

Sedentary lifestyle, the fourth leading cause of global mortality, is becoming more frequent in pediatric populations [5, 6]. “Global Recommendations on Physical Activity for Health” by the WHO state that children and youth aged 5–17 should accumulate a minimum of 60 min of moderate-to-vigorous intensity physical activity (MVPA) every day [5]. But, in Spain, only a 35.2% of children and 11.9% of adolescents (over 13 years) achieved that recommendation [7]. Different approaches for obesity treatment have been proposed over the last decades, but the evidence suggests that successful intervention does include diet and physical activity recommendations, behavioral therapy and family implication [8, 9]. Physical activity is associated with changes in body weight and body fat that ultimately affect insulin sensitivity [7, 10]. In obesity, the described expansion of adipose tissue leads to an increase in leptin levels. This hyperleptinemia has been associated with a pro-inflammatory status with deleterious effects on children’s health [11]. In regard to this, several intervention studies reported a decrease of leptin levels after aerobic PA in obese adolescents [11–13]. Thus, increased physical activity, specifically MVPA, may lead to a decrease of leptin levels [14]. To our knowledge, few studies have evaluated the effect of lifestyle interventions on changes in objectively measured PA levels in obese children at high metabolic risk [4, 15–19]. We hypothesized that a successful lifestyle intervention based on PA recommendations is able to modify BMI-SDS, metabolic parameters and objectively measured PA. Hence, the aims of this RCT were: [1] to assess the effectiveness of the two interventions (usual care vs. intensive care group) based on BMI-SDS changes (primary outcome), [2] to evaluate changes on biochemical and PA parameters (secondary outcome) between the two lifestyle interventions. Specifically, in our study we will assess: differences between the two lifestyle interventions and also changes (pre vs. post intervention) in the measured variables in each group.

## Methods

### Participants

The IGENOI (*Intervention Grupo Estudio Navarro de Obesidad Infantil*) study is a randomized control trial (NCT03147261) conducted in Pamplona, Navarra (Spain). It is a 2-year family-based lifestyle intervention program for children with abdominal obesity. Seven to sixteen year-old children were recruited from the Endocrinology Pediatric Units of the University of Navarra Clinic, Navarra’s Hospital Complex and Primary Health Care Centers in Pamplona. General inclusion criteria for enrollment was waist circumference above the 90th percentile, according to national data [20, 21]. Subjects with pre-diabetes or food intolerance, following special diets, regular alcohol consumption, major psychiatric illness, eating disorders or medical therapy were excluded. The study protocol was performed in accordance with the ethical standards laid down in the 2013 Declaration of Helsinki (Fortaleza, Brasil, October 2013) and was approved by the Ethics Committee of the University of Navarra (Reference number 044/2014). The parents and/or legal guardians and children involved in the trial received detailed explanations about the aim of the study. Informed assent was obtained from every child and all parents and/or legal guardians signed an informed consent according to the Helsinki declaration.

**Fig. 1:**
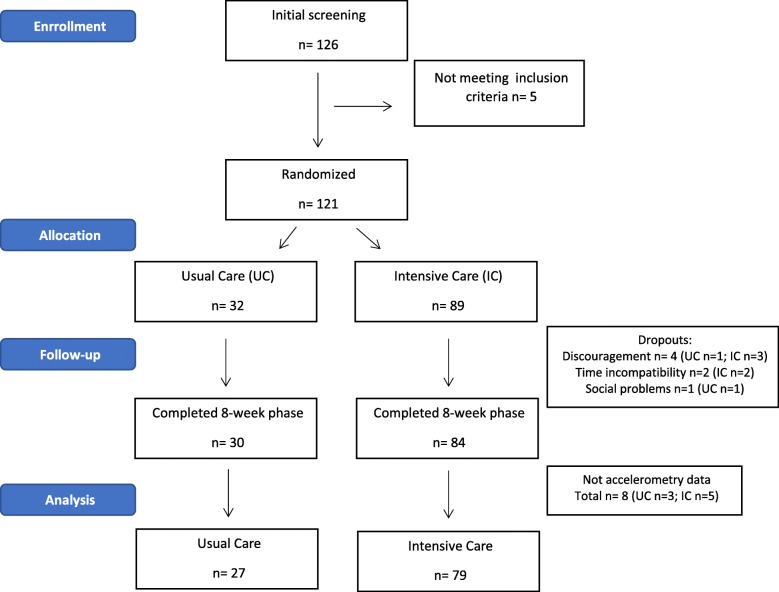
Flow chart of participants of IGENOI study

121 of the 126 recruited participants met the inclusion criteria, and 114 successfully concluded the 8-week program. The dropout rate was 6.5% and the main reasons were discouragement, social problems, inability to comply the weekly visits (school exam periods, and change of address for the notifications), as described in other trials with pediatric populations [22]. One hundred and six participants with completed data of objectively measured physical activity both at baseline and after the 8-week (Fig. 1).

The primary endpoint of the IGENOI study was to assess the effectiveness of the lifestyle interventions based on BMI-SDS values. Changes in BMI-SDS are the main outcome since successful interventions should decrease BMI-SDS near 0.5 units in order to reduce cardiometabolic risk [23]. Taking this information into consideration, sample size was estimated with the assumption of: an error of 5%, a power of 90%, a 1:3 ratio and a mean difference of 0.50 (SD 0.47) units in BMI-SDS after the lifestyle intervention. The sample size calculation indicated that 13 and 39 subjects were needed for usual care and intensive care group, respectively. The rationale for the difference in the size of both groups relies on the fact that a high number of subjects could benefit from intensive intervention as indicated in other program for obese children [24].

### Study design/ lifestyle intervention

The IGENOI study is a family-based lifestyle program carried out by a multidisciplinary team (dietitians, pediatricians, psychologist, physical activity experts, and nurses) in a clinical setting. It consists of a two-year program that comprises an 8-week phase with individual and group sessions and a follow-up period of 22 months. Our research group has different weight loss interventions where we have observed that in a short time period (8 to 10 weeks) there are substantial changes in weight loss and other metabolic parameters [8, 9]. In this study we present data from the treatment period corresponding to the first 8-weeks, since the study is still on going.

Participants were randomly assigned to the usual or intensive care group with a ratio of 1:3. The randomization was performed using a computer-generate randomization. The intensive care group was advised to follow a fully-day meal plan during the intensive phase. This diet consists on a moderate hypocaloric Mediterranean diet to not to interfere with children’s growth. Energy restriction (10 to 40%) was calculated according to obesity degree and physical activity levels, as described elsewhere [9, 25]. The dietary pattern was based on a high consumption of fruits (3 portions per day) and vegetables (2 portions per day), legumes, whole grains and olive oil; moderate consumption of dairy products, poultry and fish, and the reduction of processed and red meats, limiting them to 1 portion per week. Standard paediatric recommendations on healthy diet were given to usual care subjects. Participants of both groups were aimed to accumulate an extra time per week of 200 min of PA at a 60–75% of their maximum heart rate.

Intensive care participants and their parents received six 30 min-sessions lead by the dietitian during the 8-week period in order to monitor the accomplishment of the diet. One parallel group sessions was organized for 1) intensive care participants and 2) their parents or legal tutors. During the group sessions parents were told their role in the intervention and the obesity related comorbidities, while children were taught about different topics such as energy balance, portion sizes, groups of foods, the importance of the breakfast and physical activity [25]. On the other hand, usual care participants and their parents received one 30-min individual session with the dietitian and five monitoring visits to assess anthropometric parameters.

### Anthropometric, clinical and biochemical measurements

Anthropometric measurements (body weight, height) were evaluated by trained personnel following standard procedures. Body mass index (BMI) was calculated as weight divided by squared height (Kg/m^2^), and these values were converted into standard deviations (BMI-SDS) using age and sex-specific cut-off points according to Spanish reference growth charts [20]. Waist and hip circumferences were assessed with a non-stretchable measuring tape (Type SECA 200) following standard procedures.

Clinical outcomes such as pubertal stage (Tanner stage) and the presence of *acanthosis nigricans* were examined by pediatricians of the team [26].

Venous blood samples were obtained after an overnight fast. Glucose, insulin and lipid profiles were determined by standard autoanalyzer techniques. Homeostasis model assessment of insulin resistance (HOMA-IR) was calculated from fasting glucose and insulin values. Leptin levels were measured by ELISA (R&D Systems, Minneapolis, MN). All the measurements were taken at baseline and after the 8 week period.

### Physical activity and sleep duration

Physical activity and sedentary time were objectively assessed using triaxial accelerometry over four days, including, at least, two weekdays in all participants. Participants and parents were instructed on wearing the accelerometer (Actigraph wGT3X-BT, Actigraph LLC,Penascola,Florida, USA) around the non-dominant waist all the time, including sleep time, and removing it just for water-related activities (bathing or showering). The monitors were initialized using 60-s epochs, as described elsewhere [15].

Accelerometry data were analyzed using ActiLife 6.0 software (Actigraph LLC, Penascola, Florida, USA). Continuous 24-h accelerometer data were recorded from weekdays and weekend days and were analyzed separately. Total PA was obtained by weighting 5 times weekdays plus two times weekend days by two and dividing the result by seven, as previously reported [27]. Data were expressed as counts per minute (CPM). PA intensity was categorized using validated cut-points (Evensons) for children and adolescents. CPM were under 100 was considered sedentary time. CPM between 101 and 2295 was considered light PA. Moderate and vigorous physical activity were combined into moderate-to-vigorous PA (MVPA) when counts were over 2296 CPM.

Sleep was assessed using the accelerometer Actigraph wGT3X-BT, and the data was analysed using the Sadeh algorithm derived from fundamental research performed by Avi Sadeh et al. [28] .This algorithm was commonly used in younger adolescents [29](10 to 11 years old) .

### Statistical analysis

All statistical analyses were two-tailed and a *p* value < 0.05 was considered as statistically significant. Variables were described using mean ± SD. Student’s t test was used for the comparison between groups (two independent groups test) and for the comparison within group (paired test) before and after the intervention. The change in each variable was calculated as the difference between post- and pre- intervention values for each subject.

Analyses of covariance (ANCOVA) were performed to assess the changes in anthropometric, biochemical and PA variables between usual care and intensive care groups after the adjustment for potential confounders: the studied variable at baseline, baseline BMI-SDS, sex and Tanner stage." Stata 12.0 (StataCorp, USA) for Windows was used in all the analyses.

Furthermore, we fitted a multivariable adjusted linear regression to examine the association of changes in leptin with changes in metabolic parameters and PA intensity.

## Results

The study population includes 106 children with obesity (BMI-SDS 2.89 ± 1.05) (37.7% boys), with waist circumference over the sex and age-specific 90th percentile and a mean age of 11.31 ± 2.47 years old. At baseline, participants spent, on average, 997.9 (101.6) min/day on sedentary activities, including sleep time, 43.9 (23.4) min/day in MVPA activities, and 394.2 (95.9) min/day on light PA.

Most abdominal obese children (74.5%) do not fulfilled the WHO recommendations of being more than 60 min/day on MVPA at baseline. Participants were more active during weekdays compared to weekend days (Additional file 1).

### Effectiveness of the lifestyle intervention

As expected, due to the randomization, participants in the two groups were similar for most of the clinical parameters at baseline, except for blood glucose levels (Table 1). The usual care group (*n* = 27) had significantly higher glucose levels compared with the intensive care group (*n* = 79) (*p* = 0.016). We did not find significant differences between groups regarding age, sex or Tanner stage.

**Table 1:** Changes in anthropometric and biochemical measures after the lifestyle intervention in children with abdominal obesity (*N* = 106)

	Usual care group (*n* = 27)			Intensive care group (*n* = 79)			*P* ^2^
	Baseline	8 week	P^1^	Baseline	8 week	*P* ^1^	
Age	10.74 (2.39) 33/67			11.50 (2.48) 39/61			
Sex (male/female) (%)							
Tanner Stage (I,II,III,IV,V) (%)	37.4/ 4.1/ 29.16/ 4.1/ 25			32.9/21.1/14.4/6.6/25			
Waist circumference (cm)	86.97 (11.53)	82.54 (10.51)	**< 0.001**	86.30 (10.98)	82.37 (11.15)	**< 0.001**	0.926
Weight (Kg)	64.81 (17.32)	62.92 (16.41)	**< 0.001**	66.96 (19.21)	64.29 (19.13)	**< 0.001**	0.057
Height (cm)	149.28 (12.64)	150.47 (12.66)	**< 0.001**	151.77 (13.18)	152.63 (13.03)	**< 0.001**	0.063
BMI (Kg/m^2^)	28.54 (4.35)	27.31 (4.09)	**< 0.001**	28.40 (4.46)	26.91 (4.62)	**< 0.001**	0.075
BMI-SDS	3.07 (1.24)	2.62 (1.29)	**0.002**	2.83 (0.98)	2.32 (1.03)	**< 0.001**	**0.016**
Hip circumference (cm)	98.34 (12.02)	96.71 (10.92)	**0.012**	99.07 (12.77)	96.42 (13.37)	**< 0.001**	**0.024**
Waist to hip ratio	0.88 (0.07)	0.85 (0.06)	**< 0.001**	0.87 (0.06)	0.85 (0.06)	**< 0.001**	0.066
Waist to height ratio	0.58 (0.05)	0.54 (0.05)	**< 0.001**	0.56 (0.04)	0.53 (0.04)	**< 0.001**	0.971
Glucose (mg/dL)	92.18 (6.45)†	86.40 (5.09)	**< 0.001**	88.10 (6.08)†	85.59 (6.28)	**0.004**	0.738
Insulin (μU/mL)	20.34 (20.29)	17.93 (15.35)	0.329	15.30 (7.15)	13.09 (6.01)	**0.010**	0.147
HOMA-IR	4.65 (4.82)	3.92 (3.35)	0.271	3.37 (1.73)	2.80 (1.33)	**0.006**	0.187
Leptin (ng/mL)	38.82 (19.24)	23.49 (14.95)	**< 0.001**	30.58 (15.22)	22.35 (20.63)	**< 0.001**	0.505

Values are means (SD) or %. Abdominal obesity was defined as WC above the sex and age-specific 90th percentile. † Baseline differences p = 0.016; P^1^ paired ttest for the group comparison after intervention program; The change in each variable was calculated as the difference between post- and pre- intervention values for each subject (usual care or intensive care subjects); P^2^ is for the comparison between groups (usual care vs. intensive care) of the mean change in each variable adjusted for the corresponding variable at baseline, baseline BMI-SDS, sex and Tanner stage

Abbreviations: *BMI* Body mass index, *BMI-SDS* Standard deviation score for body mass index

*P* values below 0.05 are written in boldface

The decrease in BMI-SDS (*p* = 0.029) and hip circumference (*p* = 0.019) was significantly higher in the intensive care group compared to the usual care group after adjusting for potential confounders (Table 1). Notably, participants in both groups achieved a significant reduction in waist circumference (Δ – 4.42 cm for usual care vs. Δ − 3.93 cm for intensive care group).

Regarding biochemical parameters, no differences were found between the two lifestyle interventions. A reduction in glucose (*p* = 0.004), insulin (*p* = 0.010), and leptin (*p* < 0.001) levels were observed in the intensive care group. Meanwhile, subjects in the usual care group did also significantly decrease glucose (*p* < 0.001) and leptin (*p* < 0.001) levels.

### Changes in physical activity after the lifestyle intervention

As this is a randomized study, no differences were found on PA levels at baseline between groups (Additional file 2). No differences were found between the two lifestyle interventions in PA levels (Table 2). Interestingly, light PA significantly decreased in both the intensive and usual care group after the intervention. Participants from the intensive care group MVPA significantly increased from 43.5 min to 49.1 min (*p* = 0.024) in the intensive care group (Table 2). Interestingly, when all participants were analyzed in a multivariable-adjusted model, an inverse association between the percentage of change in MVPA and the percentage of change in leptin levels was found (*B*: -2.17; 95% CI: -3.73 to − 0.61). Moreover, changes in leptin were also associated with changes in anthropometric and metabolic parameters (Table 3).

**Table 2:** Objectively measured physical activity before and after the lifestyle intervention in children with abdominal obesity

	Usual care group (*n* = 27)			Intensive care group (*n* = 79)			*P* ^2^
	Baseline	8-week	*P* ^1^	Baseline	8-week	*P* ^1^	
CPM	641.75 (183.23)	589.37 (233.69)	0.257	569.60 (181.93)	577.64 (203.18)	0.666	0.348
Sleep time (min)	510.33 (65.76)	516.62 (141.58)	0.825	538.51 (82.52)	526.42 (72.53)	0.344	0.814
Sedentary PA (min)	456.31 (114.48)	490.79 (160.13)	0.285	467.71 (135.22)	495.33 (117.83)	0.119	0.971
LPA (min)	420.34 (79.17)	369.69 (110.41)	**0.003**	385.34 (99.93)	361.55 (96.26)	**0.040**	0.668
MVPA (min)	45.17 (22.98)	44.89 (25.26)	0.955	43.56 (23.79)	49.08 (23.90)	**0.024**	0.217
Steps (number)	10,540 (3105)	9953 (3774)	0.391	10,151 (3083)	10,288 (2981)	0.740	0.332

Numbers are means (SD). Abdominal obesity was defined as WC above the sex and age-specific 90th percentile. P^1^ paired ttest for the group comparison after the intervention program; The change in each variable was calculated as the difference between post- and pre- intervention values for each subject (usual care or intensive care subjects); P^2^ is for the comparison between groups (usual care vs. intensive care) of the mean change in each variable adjusted for the corresponding variable at baseline, baseline BMI-SDS, sex and Tanner stage

Abbreviations: *CPM* Counts per minute, *LPA* Light physical activity, *MVPA* Moderate-to-vigorous physical activity, *PA* Physical activity

*P* values below 0.05 are written in boldface

**Table 3:** Association between changes in leptin levels with changes in metabolic and PA parameters: multivariable linear regression analysis in children with abdominal obesity

	% Δ leptin			
	Crude regression		Adjusted regression^1^	
	β	*P*	β	*p*
% Δ BMI-SDS	0.176	**< 0.001**	0.146	**0.001**
% Δ waist circumference	0.040	**< 0.001**	0.051	**< 0.001**
% Δ Waist-to-height ratio	0.041	**< 0.001**	0.050	**< 0.001**
% Δ glucose	0.009	0.639	0.040	0.077
% Δ insulin	0.194	0.049	0.244	0.055
% Δ HOMA-IR	0.187	0.077	0.271	**0.050**
% Δ Sleep time	−0.035	0.573	−0.119	0.083
% Δ Sedentary PA	0.003	0.973	0.111	0.241
% Δ LPA	0.020	0.670	0.001	0.994
% Δ MVPA	−0.088	0.888	−2.173	**0.007**

Abdominal obesity was defined as WC above the sex and age-specific 90th percentile. ^1^ The regression model was adjusted for the corresponding variable at baseline, BMI-SDS, sex and Tanner stage

Abbreviations: *BMI-SDS* Standard deviation score for body mass index, *LPA* Light physical activity, *MVPA* Moderate-to-vigorous physical activity, *PA* Physical activity

*P* values below 0.05 are written in boldface

## Discussion

In this study the two lifestyle interventions reduced anthropometric indexes and lowered light PA in abdominal obese children. No significant differences were observed between intensive care and usual care in PA levels. Intensive care participants significantly increase MVPA levels after the intervention. Moreover, changes in MVPA were inversely associated with changes in leptin levels after the intervention.

Concerning objectively measurement PA levels, our participants spent 44 min/day on MVPA at baseline. These data are similar to those found in overweight and obese pediatric populations from Madrid, Spain (9 years old: 58.6 min/day in MVPA, and 15 years old: 49.3 min/day) [7]. Both studies reported an appropriate management of the accelerometers concerning the cut-off points and epoch rate used. It is worth mentioning that our participants were 11.31 years old and had abdominal obesity.

Evidence shows that PA levels are different between normal-weight and obese subjects. Besides, PA levels are higher in children than in adolescents and in boys than in girls, with accelerometer-measured MVPA ranging from 49.1 to 85.1 min/day in normal-weight children and adolescents [7, 30–32].

We addressed the achievement of WHO recommendations on MVPA levels in our population. Only 25% of participants accumulated more than 60 min/day of MVPA at baseline. When considering the day of the week, 32% of the participants achieved the recommendations during the weekdays but only 16% of them did it at weekends. The evidence regarding this point is controversial. Some authors recommend the promotion of PA during the weekdays because children have PA lessons at the school [33, 34], while many others have observed that children are more physically active during the weekends, probably due to the fact that they have more spare time [7, 35] at the weekends. In our study, we observed that abdominal obese children are more physically active during the weekdays and more sedentary during the weekends. This could be explained because during the weekdays children attend school-based activities and sport games after the classes as well. However, in the weekends they may not have that many organized activities, the opportunities of being physically active are less and thus they spend more time in sedentary activities [36].

In the literature we found several lifestyle intervention studies that examined both adiposity indexes (i.e. BMI-SDS) and objectively measured PA levels [15, 16, 18] in obese children. First, a family-based behavioral program in 210 families of obese children achieved a reduction in − 0.1 units of BMI-SDS but did not report changes in MVPA after 12-week intervention [15]. Another study performed in 41 Latino families of obese children did not observe changes in either BMI-SDS or MVPA levels after a 6 months intervention [16]. In contrast, Hughes et al. (2008) performed a controlled trial with an intervention based on a traffic light diet and advice to increase PA on 1 h per day [18]. They found significant changes in sedentary behavior and light PA after 6 months of follow-up in the intervention group, but no changes were found in MVPA levels. Two more trials reported no changes in MVPA despite using novel approaches, such as active video games and motivational interviewing treatment [4, 17].

To our knowledge, this is the first intervention program that achieved a significantly increase in MVPA (+ 5.5 min/day,) in intensive care subjects. We did not found differences in PA between intervention groups. Intensive care subjects were under a moderate hypocaloric Mediterranean diet and achieved a successful weight loss (Δ BMI-SDS = − 0.51) that was higher than usual care group. It has been described that the combination of PA and Mediterranean diet might provide greater health benefits that those acquired separately in a recent meta-analysis [37]. In addition, a close follow-up of the participants (i.e number of visits and duration) and family enrolment are important issues in regard to the effectiveness of a paediatric intervention [38].

Leptin is a peptide hormone secreted by adipose tissue which plays a central role in regulating human energy homeostasis [24, 25]. Leptin levels are higher in obese adult and children, but physical activity might effectively reduce adipose tissue and lower leptin levels [26]. Obese children in this study reduced leptin levels after the intervention. Moreover, an inverse association between changes in MVPA and leptin levels was observed. Similar findings were reported in other intervention studies using objectively measured PA levels in adults [14].

The strengths of our study include: [1] the longitudinal design; [2] the effectiveness of the intervention with obese participants achieving a substantial weight loss; [3] the use of objectively measured physical activity. On the other hand, the diversity in important variables such as age and pubertal stage of the studied population is a limitation of this study. In order to control these potential confounders, sex, age and Tanner stages were included in the statistical models. Another limitation could be a possible lack of statistical power for PA analysis between the two lifestyle interventions.

## Conclusion

In conclusion, the two lifestyle interventions were successful, since a reduction in anthropometric indexes and light PA in abdominal obese children was achieved. No significant differences were observed between intensive care and usual care groups in regard to PA. Intensive care participants following a hypocaloric Mediterranean diet significantly increased MVPA. Changes in MVPA were inversely associated with changes in leptin levels after the intervention.

## Additional files

Additional file 1: **Table S1**. Physical activity characteristics measured by accelerometry before lifestyle intervention in children with abdominal obesity (*N* = 106). (DOCX 13 kb)

Additional file 2: **Table S2**. Baseline characteristics in participants with abdominal obesity divided by type of intervention. (DOCX 14 kb)

## Abbreviations

BMI: body mass index
BMI-SDS: body mass index standard deviation
CPM: counts per minute
HOMA-IR: homeostasis model assessment for insulin resistance
MVPA: moderate to vigorous physical activity
PA: physical activity
WHO: world health organization
