# Supplementary material for: Changes in objectively measured physical activity after a multidisciplinary lifestyle intervention in children with abdominal obesity: a randomized control trial
Source: BMC Pediatr. 2019 Apr 4;19:90. doi: 10.1186/s12887-019-1468-9 (PMC6448302; doi:10.1186/s12887-019-1468-9)
Supplement: Supplementary file 1 — Table S1. Physical activity characteristics measured by accelerometry before lifestyle intervention in children with abdominal obesity (N = 106). (DOCX 13 kb) [file 12887_2019_1468_MOESM1_ESM.docx]

**Supplementary Table 1**. Physical activity characteristics measured by accelerometry before lifestyle intervention in children (N = 106) with abdominal obesity.

|  | **Weekdays** | **Weekend days** | **p** |
| --- | --- | --- | --- |
| METS | 1.55 (0.20) | 1.48 (0.20) | **<0.001** |
| CPM | 607.17 (193.93) | 519.68 (194.05) | **<0.001** |
| Sedentary PA (min) | 986.97 (107.31) | 1030.72 (112.70) | **<0.001** |
| LPA (min) | 402.30 (101.59) | 374.15 (103.24) | **<0.001** |
| MVPA (min) | 48.04 (26.69) | 34.92 (25.21) | **<0.001** |
| Steps (number) | 10832 (3470) | 8795 (3894) | **<0.001** |

Values are means (SD). Abdominal obesity was defined as WC above the sex and age-specific 90^th^ percentile.

Abbreviations: CPM, counts per minute; LPA, light physical activity; MVPA, moderate-to-vigorous physical activity; PA, physical activity
