# Supplementary material for: Changes in objectively measured physical activity after a multidisciplinary lifestyle intervention in children with abdominal obesity: a randomized control trial
Source: BMC Pediatr. 2019 Apr 4;19:90. doi: 10.1186/s12887-019-1468-9 (PMC6448302; doi:10.1186/s12887-019-1468-9)
Supplement: Supplementary file 2 — Table S2. Baseline characteristics in participants with abdominal obesity divided by type of intervention. (DOCX 14 kb) [file 12887_2019_1468_MOESM2_ESM.docx]

|  | Usual Care | Intensive Care | p |
| --- | --- | --- | --- |
| **Age** | 10.74 (2.39) | 11.50 (2.48) | 0.166 |
| **Sex (male/female) (%)** | 33/67 | 39/61 | 0.585 |
| **Tanner Stage (I,II,III,IV,V) (%)** | 37.4/ 4.1/ 29.16/ 4.1/ 25 | 32.9/21.1/14.4/6.6/25 | 0.236 |
| **Waist circumference (cm)** | 86.97 (11.53) | 86.30 (10.98) | 0.788 |
| **Weight (Kg)** | 64.81 (17.32) | 66.96 (19.21) | 0.608 |
| **Height (cm)** | 149.28 (12.64) | 151.77 (13.18) | 0.395 |
| **BMI (Kg/m^2^)** | 28.54 (4.35) | 28.40 (4.46) | 0.889 |
| **BMI-SDS** | 3.07 (1.24) | 2.83 (0.98) | 0.309 |
| **Hip circumference (cm)** | 98.34 (12.02) | 99.07 (12.77) | 0.797 |
| **Waist to hip ratio** | 0.88 (0.07) | 0.87 (0.06) | 0.397 |
| **Waist to height ratio** | 0.58 (0.05) | 0.56 (0.04) | 0.213 |
| **Glucose (mg/dL)** | 92.18 (6.45) | 88.10 (6.08) | **0.016** |
| **Insulin (µU/mL)** | 20.34 (20.29) | 15.30 (7.15) | 0.193 |
| **HOMA-IR** | 4.65 (4.82) | 3.37 (1.73) | 0.170 |
| **Leptin (ng/mL)** | 38.82 (19.24) | 30.58 (15.22) | 0.183 |
| **CPM** | 641.75 (183.23) | 569.60 (181.93) | 0.078 |
| **Sleep time (min)** | 510.33 (65.76) | 538.51 ( 82.52) | 0.211 |
| **Sedentary PA (min)** | 456.31 (114.48) | 467.71 (135.22) | 0.144 |
| **LPA (min)** | 420.34 (79.17) | 385.34 (99.93) | 0.101 |
| **MVPA (min)** | 45.17 (22.98) | 43.56 (23.79) | 0.759 |
| **Steps (number)** | 10540 (3105) | 10151 (3083) | 0.573 |

**Supplementary Table 2**. Baseline characteristics in abdominal obese participants taking into account the arm of intervention.

Values are means (SD) or %. Abdominal obesity was defined as WC above the sex and age-specific 90^th^ percentile. P^1^ ttest is for the comparison between groups (usual care vs. intensive care).

Abbreviations: BMI, body mass index; BMI-SDS, standard deviation score for body mass index; CPM, counts per minute; LPA, light physical activity; MVPA, moderate-to-vigorous physical activity.
